# Early Versus Late Initiation of Endovascular Therapy in Patients with Severe Cerebral Venous Sinus Thrombosis

**DOI:** 10.1007/s12028-024-02046-7

**Published:** 2024-07-23

**Authors:** Philipp Bücke, Hans Henkes, Johannes Kaesmacher, Mirjam R. Heldner, Adrian Scutelnic, Marcel Arnold, Thomas R. Meinel, Alexandru Cimpoca, Thomas Horvath, Elina Henkes, Hansjörg Bäzner, Victoria Hellstern

**Affiliations:** 1 Department of Neurology, Inselspital, Bern University Hospital and University of Bern, Freiburgstrasse 16, 3010 Bern, Switzerland; 2 Neuroradiologische Klinik, Klinikum Stuttgart, Kriegsbergstrasse 60, 70174 Stuttgart, Germany; 3 Medical Faculty, Universität Duisburg-Essen, Hufelandstrasse 55, 45122 Essen, Germany; 4 Institute for Neuroradiology, Inselspital, Bern University Hospital and University of Bern, Freiburgstrasse 16, 3010 Bern, Switzerland

**Keywords:** Sinus thrombosis, Thrombectomy, Intracerebral hemorrhage

## Abstract

**Background:**

Endovascular therapy (EVT) for severe cerebral venous sinus thrombosis (CVST) is controversial in terms of indication and clinical benefit. The impact of delay of EVT on functional recovery is unclear. This study aimed to investigate the effect of early versus late initiation of EVT in severe CVST.

**Methods:**

From prospective EVT and CVST registries, patients with CVST diagnosed between January 2010 and December 2022 were retrospectively identified for this multicenter collaboration. EVT was considered in severe CVST with features prone to a poor prognosis. We compared early (< 24 h) with late (> 24 h) initiation of EVT after the presentation in the emergency department and subsequent CVST diagnosis. Outcome parameters included functional independence (modified Rankin Scale [mRS] score 0–2) at 90 days, mRS score at discharge, in-hospital mortality, and mortality at 3 months.

**Results:**

Of 363 patients with CVST, 45 (12.4%; 31 [early EVT] vs. 14 [late EVT]) were included in this study. We found a higher proportion of patients with functional independence at 3 months among early versus late EVT (66.7% vs. 27.3%; odds ratio [OR] 5.3; 95% confidence interval 1.02–25; *p* = 0.036). In multivariate logistic regression, late EVT was inversely correlated with functional independence (OR 0.17 [0.04–0.83]; *p* = 0.011). The mortality rate was 16.7% versus 36.4% (mRS 6 at 3 months, OR 0.34, 95% confidence interval 0.07–1.75; *p* = 0.217) at 90 days for early versus late EVT.

**Conclusions:**

We observed a higher rate of functional independence in patients with early EVT. These preliminary findings must be confirmed in subsequent randomized controlled trials evaluating a “time-is-brain” paradigm for EVT in CVST.

**Supplementary Information:**

The online version contains supplementary material available at 10.1007/s12028-024-02046-7.

## Introduction

Cerebral venous sinus thrombosis (CVST) is a rare but potentially life-threatening condition associated with intracerebral hemorrhage (ICH), cerebral venous infarction, and brain edema [1–3] CVST accounts for approximately 0.5–1% of all strokes, with a global incidence of 1.32 per 100,000 person-years, and appears to be more common in children, young adults, and women [1, 3]. In recent years, increased incidence rates have been observed as both coronavirus disease-2019 adenoviral-based vaccinations and the coronavirus disease-2019 infection itself seem to facilitate the development of CVST [4, 5].

CVST prognosis is variable, with approximately 80% of patients regaining functional independence [1–3, 6, 7]. Yet, dependency rates are estimated to be around 15% of cases, with a case-specific mortality rate between 4 and 10% [1, 6, 7]. Treatment strategies include early anticoagulation with unfractionated or low-molecular-weight heparin even in cases of ICH [8, 9]. Despite a rapid treatment initiation, several patients undergo clinical deterioration during the course of the disease [10, 11]. Endovascular therapy (EVT) was suggested in to improve clinical outcomes in this specific patient population [12–15]. Results from the only published randomized controlled trial for EVT in CVST revealed that EVT provided no benefit over standard care [16]. However, because of sample size limitations, the authors could not exclude potentially relevant treatment effects either overall or in specific subgroups. In selected cases of severe CVST, EVT remained a therapeutic option as part of a rescue strategy [16–18].

Ischemic stroke studies demonstrated that the immediate implementation of recanalization therapies leads to superior patient outcomes and improved survival rates [19–22]. By contrast, the impact of time in patients diagnosed with acute CVST remains unclear. This retrospective observational study aimed to determine the impact of early versus late initiation of EVT in patients presenting with severe CVST. We hypothesized that early initiation of EVT (within 24 h of hospital admission and CVST diagnosis) will lead to reduced mortality and increased functional independence.

## Methods

### Study Design

This was a multicenter retrospective observational cohort study analyzing data from two comprehensive stroke centers (Neurocenter, Klinikum Stuttgart, Stuttgart, Germany, and Department of Neurology, Inselspital, Bern University Hospital and University of Bern, Bern, Switzerland). This study was approved by the local institutional review boards (Ethics Commission of the State Medical Association of Baden-Württemberg [F-2012-077]; Kantonale Ethikkommission Bern [231/14]). Additional approval and patient consent was required for inclusion in the EVT registry (Stuttgart). The study was conducted in accordance with the Declaration of Helsinki. The Strengthening the Reporting of Observational Studies in Epidemiology guidelines were used to ensure appropriate reporting of the findings from this retrospective observational study [23].

### Study Population

Consecutive patients diagnosed with CVST of any etiology, evaluated by digital subtraction angiography (DSA), and that required EVT treatment between January 2010 and December 2022 were identified retrospectively from an ongoing prospective single-center EVT registry (Stuttgart) and the national stroke registry (Swiss Stroke Registry, Bern). The Swiss Stroke Registry is an obligatory registry prospectively including all patients with stroke (ischemic stroke, ICH, and CVST). Within established neurovascular networks, patients could be diagnosed and treated at the respective stroke center, or they might have been transferred from surrounding hospitals for EVT or neurointensive care [24]. There were no standard operating procedures declaring EVT mandatory in certain predefined situations. Treatment decisions were made on a case-by-case basis by an interdisciplinary team (neurology, interventional neuroradiology, and neurosurgery). In general, EVT was considered in patients who presented with a suspected poor prognosis (coma with a Glasgow Coma Scale [GCS] score < 9, ICH, involvement of deep cerebral veins, large thrombus load, and/or rapid clinical deterioration before EVT despite adequate anticoagulation therapy) as a rescue strategy. The presence of these clinical factors alone did not facilitate the decision to perform early or late EVT. This decision was made by the treatment team (emergency department, stroke unit, neurointensive care, interventional neuroradiology, and neurosurgery), required an interdisciplinary consultation and was based on course of the disease (i.e., slow or rapid deterioration), additional imaging findings (e.g., brain edema, venous infarction), and capacities. Various endovascular strategies (e.g., aspiration or stent retriever thrombectomy, stent placement, percutaneous transluminal angioplasty, rheolytic thrombectomy, or thrombus fragmentation) were used based on the decision of the interventional neuroradiologist. Stent placement was considered an option in cases of repeated and/or complete reocclusion. Strategies could be adapted and combined during the course of the procedure. All patients with confirmed CVST were initially treated with continuous unfractionated heparin (bolus; anti-Xa-activity controlled [therapeutic range: 0.3–0.7 IU/ml]) followed by either unfractionated or low-molecular weight heparin (after EVT). In cases of stent placement, an additional dual platelet aggregation inhibition was installed (aspirin at 100 mg per day plus clopidogrel at 75 mg per day or ticagrelor at 90 mg twice daily). If the patient underwent EVT within the first 24 h of diagnosis, we considered this approach as first-line rescue treatment, given the limited time interval between the initiation of medical therapy and the procedure.

Patients were eligible for further analysis if the following criteria were met: (1) the patient was hospitalized with acute symptoms that could be directly attributed to CVST; (2) CVST was revealed by acute cerebral imaging (magnetic resonance imaging or computed tomography with contrast for those with contraindications); (3) documentation of a modified Rankin Scale (mRS) score at discharge and/or during follow-up 3 months after the index event. Because CVST predominantly affects younger patient populations, we did not apply a lower age limit. Exclusion criteria included (1) performance of a DSA study that did not lead to a subsequent EVT (e.g., chronic CVST or cerebral sinus stenosis without thrombosis); (2) incidental detection of a chronic CVST by DSA (which was performed due to other conditions such as arteriovenous malformations); and (3) lack of informed consent.

To evaluate the impact of time (i.e., the time elapsed between emergency consultation/hospitalization and the initiation of treatment), patients were subdivided into two groups: (1) patients who underwent treatment within 24 h (early EVT) and (2) patients who underwent treatment more than 24 h after hospitalization and diagnosis of CVST (late EVT). Because there were no data on the impact of earlier versus later EVT treatment in this patient population, we decided to use the assumed time to treatment initiation in the only published randomized controlled trial on EVT in CVST (Thrombolysis or Anticoagulation for Cerebral Venous Thrombosis [TO-ACT]) as a cutoff [16]. Given an interquartile range (IQR) for the time from diagnosis to randomization between 0 and 1 days (patients had to be randomized within 24 h after CVST diagnosis), and a median time from randomization to EVT of 4.5 h (278 [IQR 105–724] minutes) plus an unknown time from emergency consultation until final diagnosis, the assumed time to treatment initiation would be approximately 24 h.

Outcome parameters included functional independence (mRS score 0–2) at 90 days, mortality (mRS 6) at 3 months, in-hospital mortality, and functional independence (mRS score 0–2) at discharge.

### Data Collection

Baseline characteristics (age, sex, medical history, symptom onset, physical examination, and mRS score) were extracted from the respective registries (PB and VH; both masked for the time elapsed between emergency consultation and EVT) and—if required—retrospectively completed according to emergency department admission notes, referral papers, and/or discharge letters. For the scope of this article, we distinguished between coma (GCS < 9) and somnolence/stupor (GCS 9–14). Imaging data were stored and analyzed in our picture archiving and communications system. We used the reports of the original read in order to describe imaging findings, such as ICH or subarachnoid hemorrhage (SAH), venous congestion, venous infarction, or thrombus load, as well as the number and location of the involved sinus(es). These findings were acquired retrospectively and verified in an additional read prior to this analysis (VH). Partial recanalization after EVT was defined as recanalization of one or more affected sinuses (but not all; residual thrombus material). In both centers, outcome data were recorded prospectively during scheduled follow-up consultations. A trained and specialized study nurse (trained in neuroradiology and neurology) collected the 3-month follow-up data on functional outcomes (mRS) and mortality via telephone calls (Stuttgart). Follow-up visits were scheduled 3 months after the index event in the outpatient department (Bern).

### Statistical Analysis

Numerical baseline characteristics were described using the median (± IQR) or mean (± standard deviation [SD]). Frequencies were used to present categorical parameters. Group comparisons of categorical parameters were performed using the Fisher exact *t*-test or the *χ*^2^ test. The Kruskal–Wallis test or the Mann–Whitney *U*-test were applied as appropriate to evaluate numerical (outcome) parameters. The comparison of outcome variables was reported using odds ratios (ORs) and 95% confidence intervals (CIs) with adjustment in case of significant differences in baseline characteristics. A *p* value of < 0.05 was considered statistically significant. Statistical analyses were performed using STATA/IC 13.1 for Windows (StataCorp, College Station, TX).

Multivariate logistic regression was performed in order to determine factors associated with good functional outcome. Because of sample size restrictions, we could only include a small number of covariables in the multivariate logistic regression. Besides time to EVT, we predefined coma and any hemorrhage (ICH and SAH) as the only covariables. Both were previously shown to be predictors of severity [25]. Because we expected a low absolute number of death cases, no multivariate analysis regarding mortality was conducted.

## Results

**Fig. 1:**
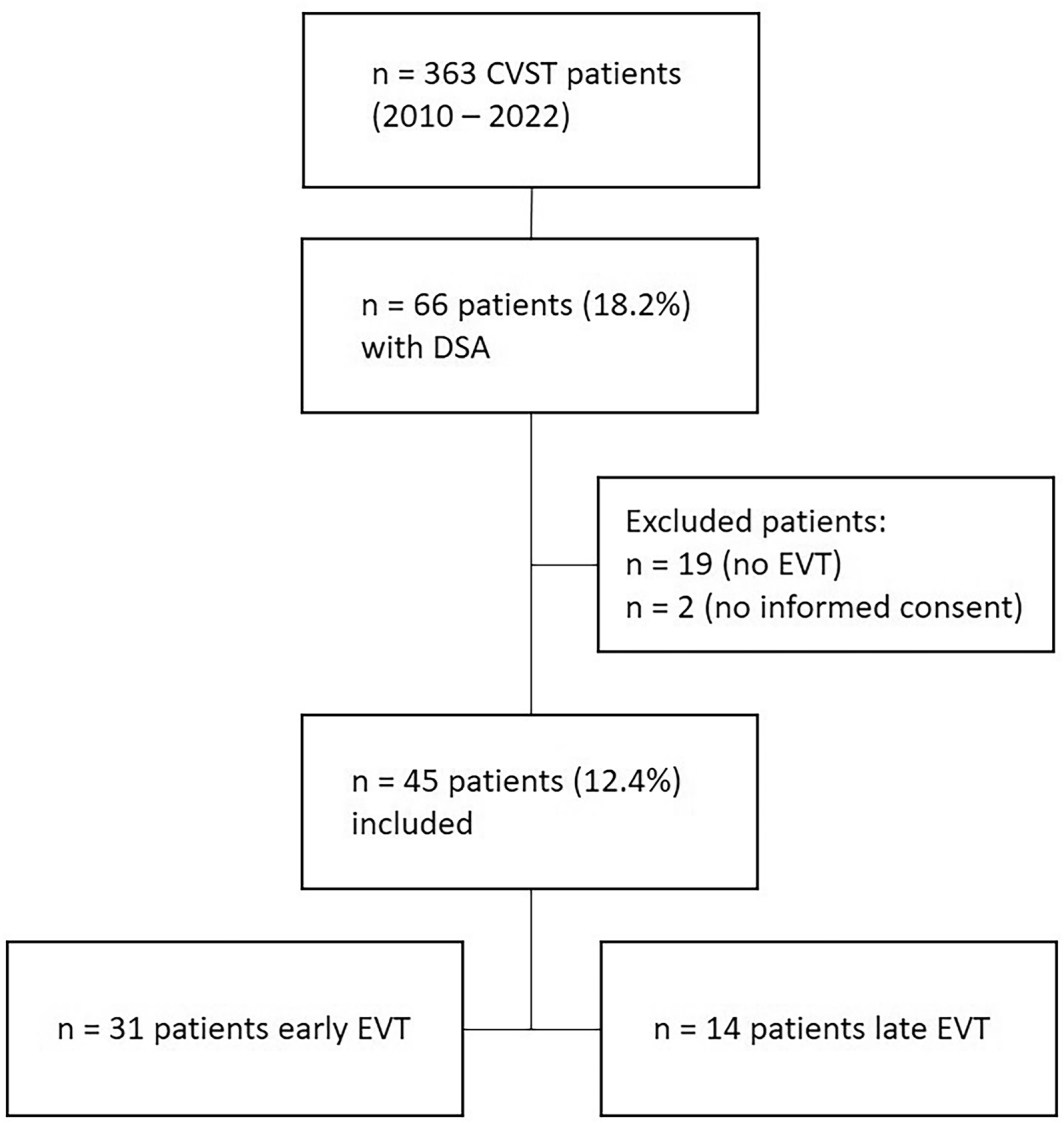
Flowchart documenting patient selection according to predefined inclusion and exclusion criteria. CVST, cerebral venous sinus thrombosis, DSA, digital subtraction angiography, EVT, endovascular therapy

Between January 2010 and December 2022, 363 patients were diagnosed with CVST. Of these, 66 patients underwent DSA (18.2%). Twenty-one patients did not meet the criteria for inclusion in this retrospective observational study: 19 patients did not undergo EVT or present with CVST as an incidental finding, and two patients did not provide informed consent (Fig. 1). We identified 45 patients (12.4%) eligible for further analysis; 31 patients in the early EVT group (underwent the procedure at < 24 h after hospitalization) and 14 patients in the late EVT group (underwent the procedure > 24 h after hospitalization) (Fig. 1). mRS scores at 90 days were available for 41 patients (30 in the early EVT group and 11 in the late EVT group).

Descriptive baseline patient characteristics, including information on CVST etiology, endovascular procedures and postprocedural medication, are shown in Table S1. There were no significant differences between the early EVT group and the late EVT group, including seizures and/or focal neurological deficits at the time of presentation (74.1% vs. 71.4%; *p* = 0.409), any impaired consciousness (somnolence/stupor or coma; 54.8% vs. 42.9%; *p* = 0.530), location and the number of affected sinuses (*p* = 0.166), deep vein involvement (25.8% vs. 7.1%; *p* = 0.236), or detection of ICH on initial imaging studies (51.7% vs. 71.4%; *p* = 0.329; see Table 1). The mean (SD) duration of the hospital stay was 12 (7) days for early EVT versus 16 (9) days for late EVT (*p* = 0.280; excluding in-hospital mortality).

**Table 1:** Comparison of baseline characteristics that may lead to a poor prognosis in patients with early versus late EVT

	Early EVT	Late EVT	p-value
Number, *n* (%)	31 (68.9)	14 (31.1)	
Demographics			
Female sex, *n* (%)	17 (54.8)	6 (42.9)	0.530
Age, mean (SD) (yr)	45.5 (19.5)	53.9 (11.1)	0.220
Clinical characteristics			
Neurological symptoms			
Headache, *n* (%)	21 (70)	9 (64.3)	0.399
PLUS: focal neurological deficit, *n* (%)	15 (48.4)	7 (50)	
PLUS: seizure, *n* (%)	7 (22.6)	1 (7.1)	
PLUS: focal neurological deficit and seizure, *n* (%)	1 (3.1)	2 (14.3)	
Level of consciousness			
Somnolence/stupor (GCS 9–14), *n* (%)	8 (25.8)	2 (14.3)	0.710
Coma (GCS < 9), *n* (%)	9 (29)	4 (28.6)	
Combined (any reduced consciousness), *n* (%)	17 (54.8)	6 (42.9)	0.530
Deterioration before EVT, *n* (%)	4 (12.9)	6 (42.8)	0.149
Imaging variables			
Number and location of the involved sinuses			
SSS, *n* (%)	22 (71)	9 (64.3)	0.166
ISS, *n* (%)	4 (12.9)	0 (0)	
TS, unilateral, *n* (%)	15 (48.4)	6 (42.9)	
TS, bilateral, *n* (%)	6 (19.4)	3 (21.4)	
SigS, unilateral, *n* (%)	15 (48.4)	7 (50)	
SigS, bilateral, *n* (%)	3 (9.7)	1 (7.1)	
RS, *n* (%)	12 (38.7)	2 (14.3)	
Large thrombus load (> 3 sinuses), *n* (%)	8 (25.8)	2 (14.3)	0.469
Involvement of deep veins, *n* (%)	8 (25.8)	1 (7.1)	0.236
Involvement of cortical veins, *n* (%)	23 (74.2)	8 (57.1)	0.256
Imaging findings at presentation			
Hemorrhagic complications, *n* (%)	19 (61.3)	10 (71.4)	0.738
ICH, *n* (%)	16 (51.7)	10 (71.4)	0.329
SAH, *n* (%)	9 (26.1)	1 (7.1)	0.137
Venous congestion, *n* (%)^a^	22 (71)	7 (50)	0.197
Venous infarction, *n* (%)	4 (12.9)	5 (35.9)	0.077
Treatment variables			
Time from hospitalization to EVT, hours, mean (SD)	9.8 (7.6)	58.1 (64.8)	< 0.001
Complete recanalization, *n* (%)	27 (87.1)	10 (71.4)	0.207
Craniectomy required, *n* (%)	3 (9.7)	3 (21.4)	0.290
Length of hospital stay (days), mean (SD)	12 (7)	16 (9)	0.280

*EVT* endovascular therapy, *SD* standard deviation, *GCS* Glasgow Coma Scale, *SSS* superior sagittal sinus, *ISS*, inferior sagittal sinus, *TS* transverse sinus, *SigS* sigmoid sinus, *RS* straight sinus, *ICH* intracerebral hemorrhage, *SAH* subarachnoid hemorrhage

^a^Defined as a focal subcortical T2 hypointensity in magnetic resonance imaging

The average time to treatment (emergency consultation/hospitalization to EVT; mean ± SD) was 24.9 ± 23.8 h. The average time elapsed between hospitalization and EVT was 9.8 ± 7.6 h for patients who underwent early and 58.1 ± 64.8 h for those who underwent late EVT (*p* < 0.001). Neurological deterioration (reduced level of consciousness, development of focal neurological deficits) was reported in 12.9% (n = 4) of those in the early treatment group and in 42.8% (n = 6) of those in the late treatment group (*p* = 0.149).

With respect to functional independence, 20/30 (66.7%) patients in the early versus 3/11 (27.3%) in the late group experienced a good functional outcome (mRS 0–2; OR 5.3; 95% CI, 1.02–25; *p* = 0.036; Table 2) at 90 days. There was no significant difference in functional independence (mRS 0–2) at discharge (15/31 [48.4%] vs. 4/14 [28.6%]; OR 2.3; 95% CI 0.6–9.1; *p* = 0.330). Our analysis revealed no significant changes in both 90-day mortality (5/30 [16.7%] in the early EVT group vs. 4/11 [36.4%] in the late EVT group; OR 0.35; 95% CI 0.07–1.75; *p* = 0.217) and in-hospital mortality (4/31 [12.9%] in early EVT vs. 4/14 [28.6%] in late EVT; OR 0.370; 95% CI 0.08–1.77; *p* = 0.370). The overall mortality rate in our cohort was 21.9% (n = 9). Outcome comparison between the two participating centers and time metrics are shown in the online supplement (supplemental material; Table S2).

**Table 2:** Outcomes after early versus late initiation of EVT

	Early EVT, *n* (%)	Late EVT, *n* (%)	OR (95% CI); *p* value
mRS 0–2 (90 days)	20 (66.7)	3 (27.3)	5.3 (1.02–25); 0.036
Mortality (90 days)	5 (16.7)	4 (36.4)	0.35 (0.07–1.75); 0.217
mRS 0–2 at discharge	15 (48.4)	4 (28.6)	2.3 (0.6–9.1); 0.330
Mortality (in-hospital)	4 (12.9)	4 (28.6)	0.37 (0.08–1.77); 0.370

*EVT* endovascular therapy, *mRS* modified Rankin Scale, *OR* Odds ratio, *CI* confidence interval

In multivariate logistic regression, there was a negative association of late EVT (treatment after 24 h; OR 0.17 [0.04–0.83]; *p* = 0.011) and functional independence at three months (Table 3). The same was observed for any baseline hemorrhage (ICH and/or SAH; OR 0.14 [0.03–0.83]; *p* = 0.028) but not for coma at initial presentation (OR 0.30 [0.07–1.26]; *p* = 0.099). These results did not change when patients with traumatic CVST were removed from the analysis (treatment after 24 h: OR 0.16 [0.03–0.72], *p* = 0.017; baseline hemorrhage: OR 0.18 [0.04–0.90], *p* = 0.037; coma: OR 0.42 [0.07–1.36], *p* = 0.122).

**Table 3:** Multivariate logistic regression model analyzing prepotential associations with good functional outcome

	OR (95% CI)	*p* value
Time from hospitalization to EVT (h)	0.17 (0.04–0.83)	0.011
Any hemorrhage (baseline)	0.14 (0.03–0.83)	0.028
Coma	0.30 (0.07–1.26)	0.099

*EVT* endovascular therapy, *OR* odds ratio, *CI* confidence interval

In term of procedural complications, we observed one postinterventional subdural hematoma in the early EVT group. Follow-up DSA (*n* = 18) detected one dural arteriovenous fistula most likely to be attributable to the intervention (early EVT).

## Discussion

The main finding of this retrospective multicenter cohort study indicates a significant increase in functional independence after early versus late EVT (66.7% vs. 27.3%) in patients with severe CVST prone to a poor prognosis. We observed a nonsignificant but potentially clinically relevant reduction in mortality (16.7% vs. 36.4%) for early EVT.

To the best of our knowledge, the question regarding a potential impact of time (meaning the time elapsed between emergency consultation/diagnosis of CVST and initiation of EVT) on functional outcomes after CVST is unanswered. Randomization in the TO-ACT trial had to occur within 24 h (from CVST diagnosis) plus an additional 278 min (median; IQR 105–724 min) from randomization to EVT [16]. There was no information on the time from emergency consultation until CVST diagnosis. Given the severity of symptoms in TO-ACT, it can be assumed that CVST was diagnosed early and within the first hours. Jedi et al. [26] reported the time from hospitalization to EVT (with a range between 0 and 7 days) in a single-center cohort without providing further analyses on a potential impact on functional outcomes. Patients included in our study were treated early (on average 24.9 h after emergency consultation). Nonetheless, there was a notable difference between the early EVT group (average interval of 9.8 h) and the late EVT group (average interval of 58.1 h).

We observed an increase in the frequency of good functional outcomes (66.7% vs. 27.3%; *p* = 0.036) for early EVT. This was regardless of the fraction of patients with features suggesting poor prognosis (e.g., impaired consciousness, 54.8% in the early EVT group vs. 42.9%; deep vein involvement, 25.8% vs. 7.1% in the late EVT group, or large thrombus load, 25.8% vs. 14.3% for late EVT). The effect of time remained significant in the multivariate logistic regression model.

Clinical deterioration prior to the intervention (e.g., a decrease in the level of consciousness, development of focal neurological symptoms) was reported more frequently in late (42.8%) as opposed to early EVT (12.9%). Although no correlation has been established in the literature, these results might be attributable to the effect of early recanalization (prior to any further brain damage). Early recanalization is assumed to be associated with less parenchymal injury and an immediate improvement of nonhemorrhagic brain damage [27]. Additional effects on long-term functional outcomes seem to be driven by an early recanalization (prior to the development of any secondary deterioration) [27, 28]. We observed a similar effect of early recanalization. Patients with late EVT did not improve over time (mRS 0–2 at discharge: 28.6%; mRS 0–2 at 3 months: 27.3%). In the early EVT group, patients did improve between discharge (48.4% with mRS 0–2) and follow-up after 3 months (66.7% with mRS 0–2).

At initial presentation, our patient population was severely affected. This may have prompted the decision to perform early EVT. A considerable number of patients presented with clinical or radiological risk factors that have been associated with a poor prognosis [10]. In addition, our patient cohort was slightly older with a higher percentage of male patients (both associated with poor functional outcomes) as compared with the literature [3, 10, 16]. This resulted in an overall mortality rate of 21.9%. Although in line with a recent case series, this is higher compared with meta-analysis data (including a higher percentage of less affected patients) [26, 29]. Although mortality rates in early EVT (16.7%) and TO-ACT (12%) were found to be comparable, increased mortality rates in our study seem to be attributable to the late EVT group (36.4%) [16].

Despite being one of the largest studies to evaluate EVT in CVST, sample size restrictions leading to a potential underestimation or misinterpretation of true treatment effects are the main limitation. Because of the retrospective study design, all associated biases apply. Based on the high percentage of patients with CVST who underwent EVT, we assume that most of the severely affected patients were provided with this treatment strategy. However, we have no outcome data on severely affected patients without EVT. Clinical deterioration prior to EVT was more frequent in the late EVT group. Although we believe that this was due to the course of the disease, together with other factors such as the presence of ICH or other medical reasons, it might have prompted the decision to perform EVT in this group. The difference in the time from emergency consultation until EVT (especially in late EVT) suggests variation between both centers (e.g., in terms of timing of the intervention). In addition, there was an imbalance in the number of patients included per center with the majority of patients (85%) being treated in one center (Stuttgart). Finally, there is a lack of information regarding the time of initial symptom onset (as a majority of patients presented with prior subacute headache lasting for several days). In most cases, the only reliable time point was the time of hospitalization. Because of the severity of symptoms, the time of the emergency department consultation most likely reflects either the onset of symptoms or an acute deterioration. Early EVT may not be early in the course of the disease, but it might be early after an acute deterioration immediately before or during hospitalization that is associated with clinical symptoms prone to a poor prognosis.

In conclusion, these preliminary results suggest a higher frequency of functional independence in early EVT as compared to late EVT in patients with severe CVST. In addition, early EVT may lead to a clinically relevant reduction in mortality. The recognition of a time-is-brain paradigm might be warranted when considering EVT for CVST in selected patients and needs to be a focus of future randomized controlled trials.

## Supplementary Information

Below is the link to the electronic supplementary material.

Supplementary file1 (DOCX 18 KB)
