# Supplementary material for: Early Versus Late Initiation of Endovascular Therapy in Patients with Severe Cerebral Venous Sinus Thrombosis
Source: Neurocrit Care. 2024 Jul 23;41(3):1047–54. doi: 10.1007/s12028-024-02046-7 (PMC11599360; doi:10.1007/s12028-024-02046-7)
Supplement: Supplementary file 1 — Supplementary file1 (DOCX 18 KB) [file 12028_2024_2046_MOESM1_ESM.docx]

| **Table S1. Description of baseline characteristics in the overall patient population and those undergoing early versus late EVT.** | | | |
| --- | --- | --- | --- |
|  | **Overall** | **Early EVT** | **Late EVT** |
| Number, N (%) | 45 (100) | 31 (68.9) | 14 (31.1) |
| Female sex, N (%) | 23 (51.1) | 17 (54.8) | 6 (42.9) |
| Age, years, mean (SD) | 48.1 (17.6) | 45.5 (19.5) | 53.9 (11.1) |
| Potential etiology, N (%) |  |  |  |
| Oral contraceptives | 6 (28.6) | 4 (25) | 2 (40) |
| Thrombophilia* | 12 (26.7) | 8 (25) | 4 (28.6) |
| Paraneoplastic | 5 (11.1) | 4 (12.9) | 1 (7.1) |
| Septic (mastoiditis, meningitis) | 2 (4.4) | 0 (0) | 2 (14.3) |
| Traumatic | 2 (4.4) | 2 (6.5) | 0 (0) |
| Headache, N (%) | 30 (68.2) | 21 (70) | 9 (64.3) |
| Refractory headache, N (%) | 25 (58.1) | 17 (58.6) | 8 (57.1) |
| Impaired consciousness, N (%) | 23 (51.1) | 17 (54.8) | 6 (42.9) |
| Number and location of the involved sinuses |  |  |  |
| SSS, N (%) | 31 (68.9) | 22 (71) | 9 (64.3) |
| ISS, N (%) | 4 (8.9) | 4 (12.9) | 0 (0) |
| TS, unilateral, N (%) | 21 (46.7) | 15 (48.4) | 6 (42.9) |
| TS, bilateral, N (%) | 9 (20) | 6 (19.4) | 3 (21.4) |
| SigS, unilateral, N (%) | 22 (48.9) | 15 (48.4) | 7 (50) |
| SigS, bilateral, N (%) | 4 (8.9) | 3 (9.7) | 1 (7.1) |
| RS, N (%) | 14 (31.1) | 12 (38.7) | 2 (14.3) |
| Large thrombus load (>3 sinuses), N (%) | 10 (22.2) | 8 (25.8) | 2 (14.3) |
| Cortical veins involved, N (%) | 31 (68.9) | 23 (74.2) | 8 (57.1) |
| Deep veins involved, N (%) | 9 (20) | 8 (25.8) | 1 (7.1) |
| Venous congestion, N (%) † | 29 (64.4) | 22 (71) | 7 (50) |
| Venous infarction at presentation, N (%) | 9 (20) | 4 (12.9) | 5 (35.9) |
| Hemorrhagic complications presentation, N (%) | 29 (64.4) | 19 (61.3) | 10 (71.4) |
| ICH, N (%) | 26 (57.8) | 16 (51.7) | 10 (71.4) |
| SAH, N (%) | 10 (22.3) | 9 (29.6) | 1 (7.1) |
| Deterioration before EVT, N (%) | 10 (22.2) | 4 (12.9) | 6 (42.8) |
| Time from hospitalization to EVT, hours, mean (SD) | 24.9 (23.8) | 9.8 (7.6) | 58.1 (64.8) |
| Medication during/after EVT, N (%) |  |  |  |
| LMWH, N (%) | 38 (84.4) | 29 (93.5) | 9 (64.3) |
| UFH, N (%) ‡ | 8 (17.8) | 3 (9.7) | 5 (35.7) |
| Aspirin, N (%) | 8 (17.8) | 7 (22.6) | 1 (7.1) |
| DAPT, N (%) | 4 (8.9) | 3 (9.7) | 1 (7.1) |
| Type of Endovascular Therapy (EVT) |  |  |  |
| Aspiration, N (%) | 44 (97.8) | 31 (100) | 13 (92.9) |
| Stent retriever, N (%) | 21 (46.7) | 16 (51.6) | 5 (35.7) |
| PTA, N (%) | 15 (33.3) | 12 (38.7) | 3 (21.4) |
| Rheolytic thrombectomy, N (%) | 14 (31.1) | 12 (38.7) | 2 (14.3) |
| Thrombus fragmentation, N (%) | 11 (24.4) | 5 (16.1) | 6 (42.9) |
| Fogarty catheter, N (%) | 1 (2.2) | 1 (3.2) | 0 (0) |
| Stent placement, N (%) | 4 (8.9) | 3 (9.7) | 1 (7.1) |
| Complete recanalization, N (%) | 37 (82.2) | 27 (87.1) | 10 (71.4) |
| Craniectomy, N (%) | 6 (13.3) | 3 (9.7) | 1 (21.4) |
| * Hereditary thrombophilia, Factor-V-Leiden, G20210A, Factor VIII mutation, PF4, IPT.  † defined as a focal subcortical T2 hypointensity in magnetic resonance imaging (MRI)  ‡ one patient switched from UFH to LMWH within the first hours after EVT  Abbreviations: EVT, endovascular therapy; N, number; SD, standard deviation; SSS, superior sagittal sinus; ISS; inferior sagittal sinus; TS, transverse sinus; SigS, sigmoid sinus; RS, straight sinus; ICH, intracerebral hemorrhage; SAH, subarachnoid hemorrhage; LMWH, low-molecular-weight heparin; UFH, unfractionated heparin; DAPT, dual antiplatelet aggregation inhibition; PTA, percutaneous transluminal angioplasty; IQR, interquartile range. | | | |

| **Table S2. Outcome Stuttgart versus Bern** | | |
| --- | --- | --- |
|  | **Early EVT;** N (%) | **Late EVT;** N (%) |
| Stuttgart | 27 (69.2) | 12 (30.8) |
| Bern | 4 (66.7) | 2 (33.3) |
| **Time from hospitalization to EVT;** hours, mean (SD) | | |
| Stuttgart | 9.7 (7.5) | 41.6 (12.1) |
| Bern | 11.3 (8.3) | 157 (131) |
| **Outcome data Stuttgart** | | |
| mRS 0-2 (90 days) | 18 (69.2) | 3 (33.3) |
| mRS 6 (90 days) | 3 (11.5) | 4 (44.4) |
| mRS 0-2 (discharge) | 14 (51.9) | 4 (33.3) |
| **Outcome data Bern** | | |
| mRS 0-2 (90 days) | 2 (50) | 0 (0) |
| mRS 6 (90 days) | 2 (50) | 0 (0) |
| mRS 0–2 (discharge) | 1 (25) | 0 (0) |
| Abbreviations: EVT, endovascular therapy; mRS, modified Rankin Scale; n, number; SD, standard deviation. | | |
